# Circulating microRNA signature as liquid-biopsy to monitor lung cancer in low-dose computed tomography screening

**DOI:** 10.18632/oncotarget.5210

**Published:** 2015-10-06

**Authors:** Stefano Sestini, Mattia Boeri, Alfonso Marchiano, Giuseppe Pelosi, Carlotta Galeone, Carla Verri, Paola Suatoni, Nicola Sverzellati, Carlo La Vecchia, Gabriella Sozzi, Ugo Pastorino

**Affiliations:** ^1^ Unit of Thoracic Surgery, Fondazione IRCCS Istituto Nazionale Tumori, Milan, Italy; ^2^ Unit of Tumor Genomics, Department of Experimental Oncology, Fondazione IRCCS Istituto Nazionale Tumori, Milan, Italy; ^3^ Unit of Radiology, Fondazione IRCCS Istituto Nazionale Tumori, Milan, Italy; ^4^ Department of Pathology and Laboratory Medicine, Fondazione IRCCS Istituto Nazionale Tumori, Milan, Italy; ^5^ Department of Clinical and Biomedical Sciences Luigi Sacco, University of Milan, Milan, Italy; ^6^ Department of Statistics and Quantitative Methods, Division of Biostatistics, Epidemiology and Public Health, Laboratory of Healthcare Research and Pharmacoepidemiology, University of Milano-Bicocca, Milan, Italy; ^7^ Department of Clinical Sciences, Section of Radiology, University of Parma, Milan, Italy; ^8^ Department of Clinical Sciences and Community Health, University of Milan, Milan, Italy

**Keywords:** lung cancer, microRNA, liquid biopsy, LDCT screening, prognosis

## Abstract

Liquid biopsies can detect biomarkers carrying information on the development and progression of cancer. We demonstrated that a 24 plasma-based microRNA signature classifier (MSC) was capable of increasing the specificity of low dose computed tomography (LDCT) in a lung cancer screening trial. In the present study, we tested the prognostic performance of MSC, and its ability to monitor disease status recurrence in LDCT screening-detected lung cancers.

Between 2000 and 2010, 3411 heavy smokers enrolled in two screening programmes, underwent annual or biennial LDCT. During the first five years of screening, 84 lung cancer patients were classified according to one of the three MSC levels of risk: high, intermediate or low. Kaplan-Meier survival analysis was performed according to MSC and clinico-pathological information. Follow-up MSC analysis was performed on longitudinal plasma samples (*n* = 100) collected from 31 patients before and after surgical resection.

Five-year survival was 88.9% for low risk, 79.5% for intermediate risk and 40.1% for high risk MSC (*p* = 0.001). The prognostic power of MSC persisted after adjusting for tumor stage (*p* = 0.02) and when the analysis was restricted to LDCT-detected cases after exclusion of interval cancers (*p* < 0.001). The MSC risk level decreased after surgery in 76% of the 25 high-intermediate subjects who remained disease free, whereas in relapsing patients an increase of the MSC risk level was observed at the time of detection of second primary tumor or metastatic progression.

These results encourage exploiting the MSC test for lung cancer monitoring in LDCT screening for lung cancer.

## INTRODUCTION

Lung cancer is the deadliest cancer worldwide, accounting for almost 20% of such fatalities [1, 2]. Lung tumors are typically asymptomatic in their early stages and are often diagnosed too late, thus failing in successful treatment. Considering that 5-year survival for stage IA patients is over 70% it appears clear how advances in early detection are crucial to enable timely curative surgery [3, 4].

In the last two decades, several lung cancer screening programs based on low-dose computed tomography (LDCT) were launched worldwide. A significant reduction in lung cancer mortality was reported among the subjects enrolled in the LDCT arm of the National Lung Screening Trial (NLST) when compared to the chest X-ray arm [5], along with a false positive rate of 96.4% and an overdiagnosis global rate of 18.5%, reaching 78.9% for indolent cancers in the LDCT arm [6]. While waiting for the results of the Dutch-Belgian Randomized Lung Cancer Screening trial (NELSON) trial [7], other smaller randomized LDCT studies did not show similar reduction in mortality [8–10], raising further concern on the generalizability of the NLST results to different screening settings [11].

Several studies are currently investigating the value of complementary non-invasive biomarkers for risk stratification, to improve cost-benefit ratio and possibly mortality reduction in LDCT screening. MicroRNAs (miRNAs) are small non-coding RNAs that specifically repress translation of target mRNAs and whose altered expression is associated with a variety of physiological processes and diseases including cancer [12]. MiRNAs are released into the bloodstream by the tumor and its microenvironment and are stable, given that they mostly circulate within exosomes or bound to specific proteins (i.e. Ago2) which protect them from RNase degradation [13–16]. Their reliability to detect cancer through a minimally invasive liquid biopsy makes miRNAs highly promising biomarkers to be employed in clinical settings [17]. We recently described, in a LDCT-based lung cancer screening trial, the diagnostic and prognostic performance characteristics of a circulating miRNA signature risk classifier (MSC test) for the early detection of lung cancer and its ability to reduce the false positive rate of LDCT from 19.7% to 3.7% [18].

In the present study, we evaluated the prognostic performance of the MSC test, as well as its ability to monitor the disease status and recurrence in lung cancer patients identified in LDCT screening programs with a total follow up of 33402 person-years. The MSC test was employed to analyze longitudinally-collected plasma samples obtained from patients before and after surgical resection of primary lung tumors.

## RESULTS

### Characteristics of subjects

The characteristics of the 3411 subjects enrolled in the pilot study (30.3%) and in the MILD screening trial with annual (34.9%) or biennial LDCT (34.8%) are reported in Table 1 according to age, sex, and tobacco habits. Mean age, gender, and smoking pack years were similar across the two studies considered, while the percentage of current smokers enrolled was higher in the pilot study (87% vs. 69%). A total of 111 subjects (3.3%) out of 33402 person-years follow-up, developed lung cancer within the first 5 years of screening, with 25 months median time from enrollment to diagnosis. For 84 (75.7%) of them, plasma samples were available to perform the MSC test.

**Table 1:** Baseline characteristics of lung cancer screening participants and information on lung cancer patients by study arm

	Pilot study Annual CT	MILD Annual CT	MILD Biennial CT
Starting date	2000	2005	2005
No. participants	1035	1190	1186
Total Follow up (person-year)	13406	10016	9980
Age, mean ± SD	58.5 ± 5.6	58.1 ± 6.0	58.1 ± 5.8
Male, *N* (%)	740 (71%)	814 (68%)	812 (68%)
Current smokers, *N* (%)	901 (87%)	820 (69%)	809 (68%)
Pack-years of cigarettes,Median (IQR)	40 (28)	39 (20)	39 (19)
Patients at 5 years, *N*	41	42	28
Histology: adenocarcinoma	29	24	19
other types	12	18	9
Patients with plasma MSC, *N*	18	40	26

MILD: Multicentric Italian Lung Detection trial; SD: standard deviation; IQR: interquartile range; MSC: miRNA signature classifier.

Ninety-nine (89.2%) lung cancers were detected as part of scheduled LDCT screening, 65 (58.6%) in stage I and 46 (41.4%) in stages II–IV. Twelve (10.8%) subjects developed lung cancer in the interval between two rounds of screening, all in stages II–IV indicating the significantly more advanced stage of non-LDCT-detected lung tumors (*p* < 0.001). Similar distributions were observed in the subset of 84 patients suitable for MSC analysis (Table 2).

**Table 2:** Distribution of lung cancer patients according to MSC at time of diagnosis

	All patients (*N* = 111)	MSC (*N* = 84)			*P* for comparison
		Low (*N* = 9)	Intermediate (*N* = 36)	High (*N* = 39)	
LDCT-detected					
Yes	99 (89.2%)	8 (9.5%)	33 (39.3%)	35 (41.7%)	1.0
No	12 (10.8%)	1 (1.2%)	3 (3.6%)	4 (4.7%)	
Stage					
I	65 (58.6%)	5 (5.9%)	27 (32.1%)	17 (20.2%)	0.04
II–IV	46 (41.4%)	4 (4.8%)	9 (10.7%)	22 (26.3%)	

MSC: miRNA signature classifier; LDCT: low-dose computed tomography

### MSC results according to clinico-pathological information

The associations between MSC at diagnosis and clinico-pathological features are summarized in Table 2. The high risk (*n* = 39), intermediate (*n* = 36) or low (*n* = 9) risk MSC groups were similarly distributed across the LDCT-detected tumors and the interval cancers (*p* = 1.0). A significant association between MSC and tumor stage was observed, since the low and the intermediate MSC risk groups were mostly composed by stage I tumors (55.6% and 75.0%, respectively) and the high risk group included 43.6% stage I tumors (*p* = 0.04).

### Survival according to clinico-pathological characteristics

**Figure 1:**
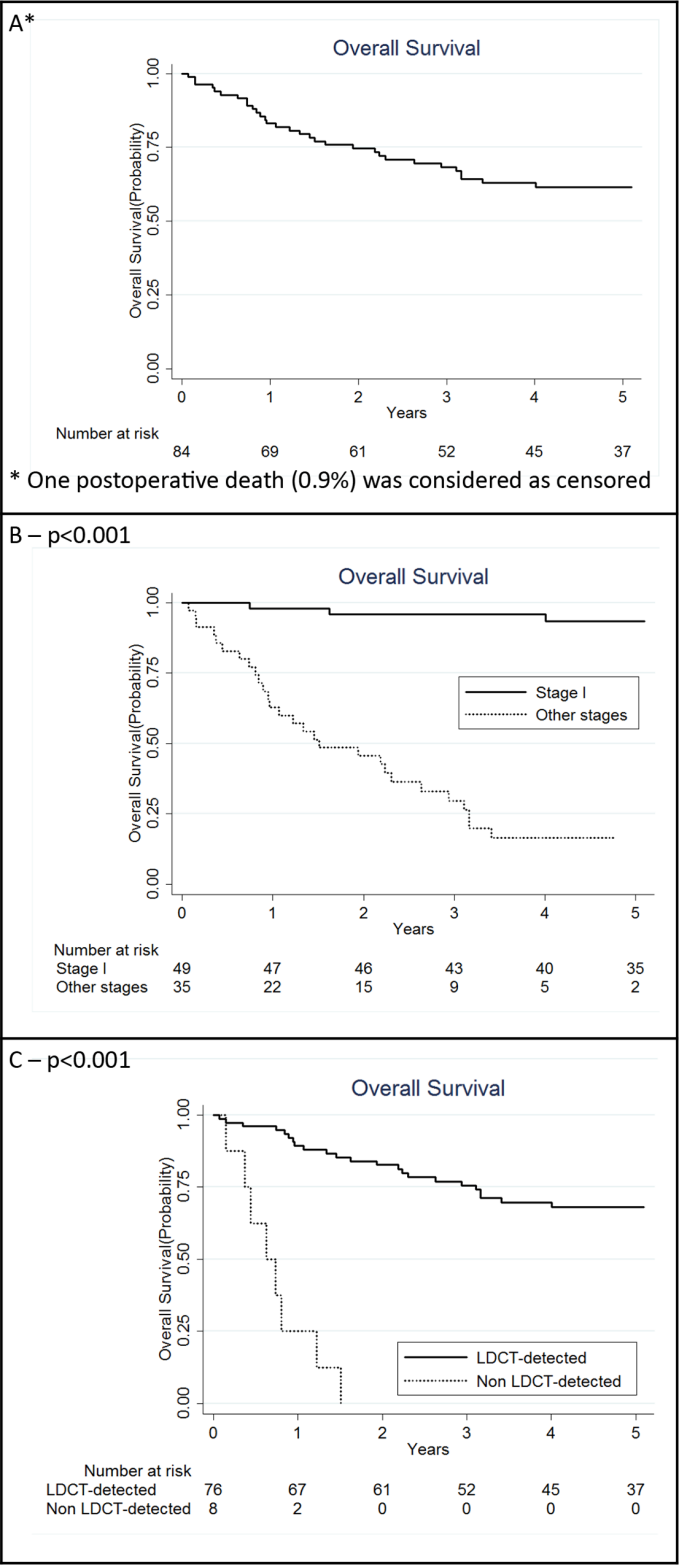
Kaplan Meier curves for overall lung cancer patients with a miRNA signature classifier (MSC) (*N* = 84) A. also in strata of clinical characteristics: stage I and other stages together B. low-dose computed tomography (LDCT)-detected and non LDCT-detected C. *P* for log rank test

The 5-year overall survival of the 84 patients was 61.6% (95% CI: 50.0%–71.3%, Figure 1A). Pathologic tumor stage was the most robust prognostic factor, with a 93.4% 5-year overall survival for stage I (median survival nc) and 8.2% for stage II–IV (median survival 1.5 yrs, *p* < 0.001, Figure 1B). While the 5-year overall survival was 68.2% (95% CI: 56.0%–77.6%) in LDCT-detected cases, all the subjects with the interval cancers survived less than 2 years (median survival 0.6 years, *p* < 0.001, Figure 1C).

### Integration of MSC results with clinico-pathological characteristics

**Figure 2:**
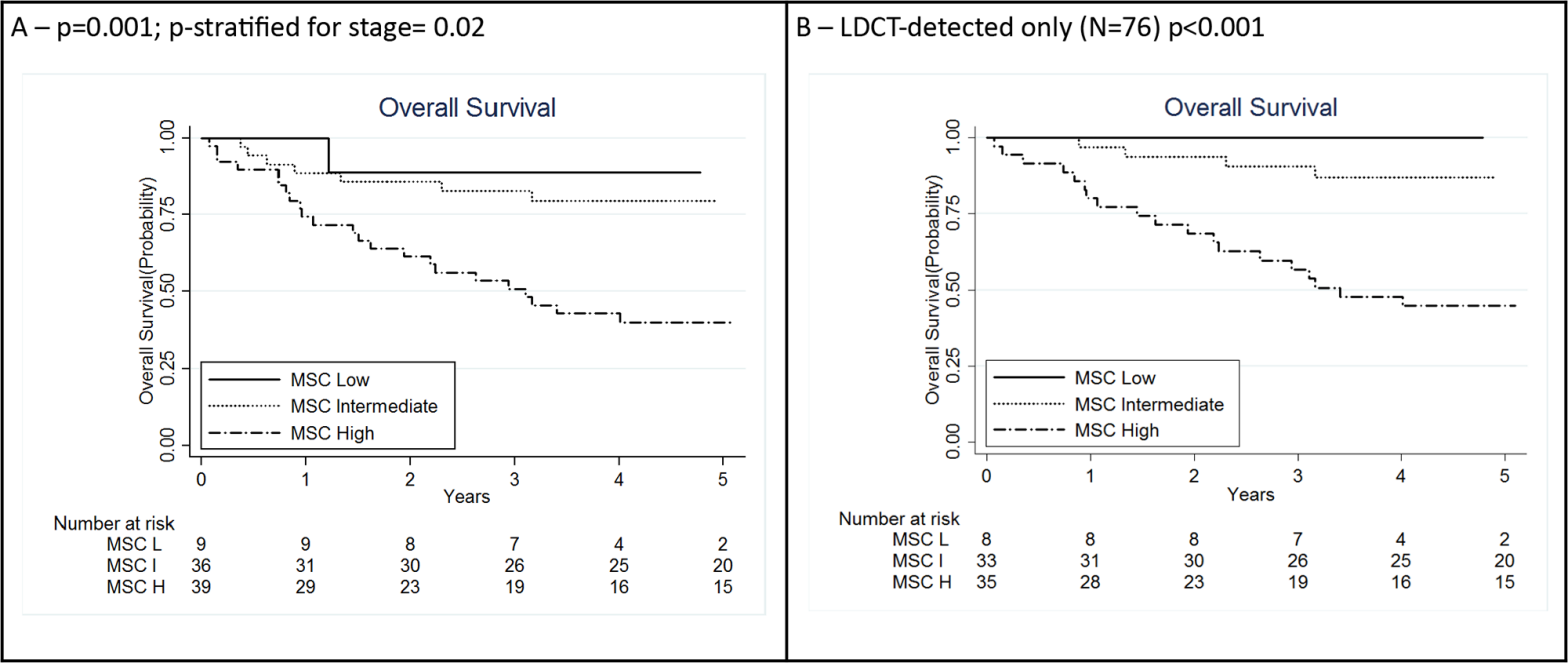
Kaplan Meier curves for lung cancer patients according to integration of miRNA signature classifier (MSC) analysis (*N* = 84) A. and clinical and pathological information: MSC analysis among low-dose computed tomography (LDCT)-detected only (*N* = 76) B. *P* for log rank test for trend. H: high; I: intermediate; L: low

Five-year survival was respectively 88.9% (95% CI: 43.3%–98.4%) for low risk MSC, 79.5% (95% CI: 61.6%–89.7%) for intermediate risk MSC and 40.1% (95% CI: 24.7%–55.1%) for high risk MSC (*p* = 0.001, Figure 2A). In the low risk group only one death was observed in a patient who developed a stage IV interval cancer. After adjusting for stage, the impact of MSC on survival was still significant (*p* = 0.02).

The prognostic value of MSC was maintained when the analysis was restricted to LDCT-detected cases with 5-years survival 100% for low risk MSC, 86.9% (95% CI: 68.8%–94.9%) for intermediate risk MSC and 44.7% (95% CI: 27.8%–60.3%) for high risk MSC (*p* < 0.001, Figure 2B).

### Monitoring lung cancer patients using MSC

**Figure 3:**
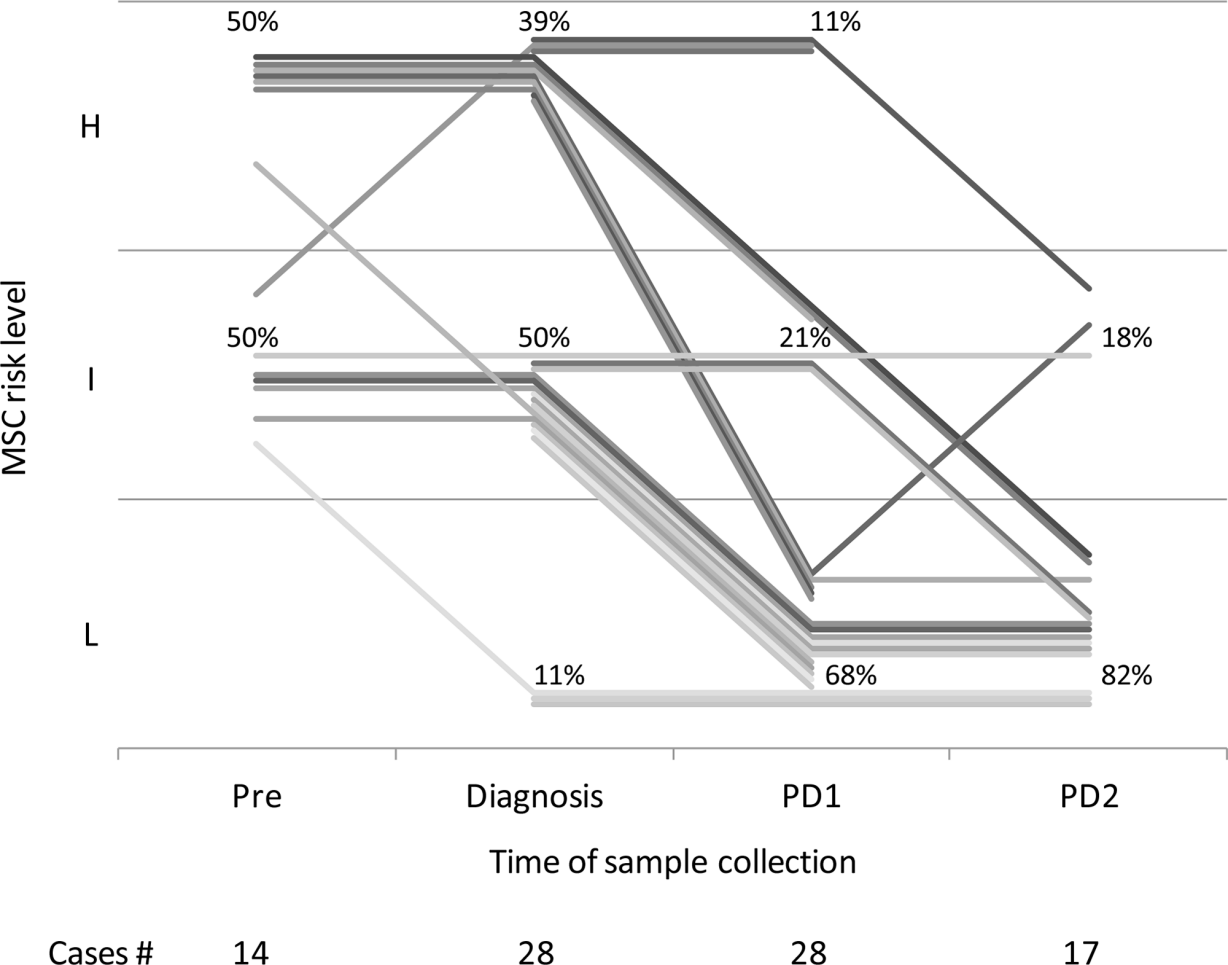
miRNA signature classifier (MSC) of 28 patients pre-diagnosis (Pre, median time from diagnosis = 1.1 years, IQR = 1.0), at diagnosis and remaining disease-free after curative surgery, at two post-operative time points: PD1 (median time from diagnosis = 1.6 years, IQR = 1.4) and PD2 (median time from diagnosis = 4.0 years, IQR = 3.3). H: high; I: intermediate; L: low

To evaluate MSC modulation during follow-up, longitudinal plasma samples (*n* = 100) before and after curative surgery were analyzed in a subset of 31 out of 44 (70.5%) alive patients (28 disease-free and 3 relapsing patients) of the MILD trial. At time of diagnosis, 11 of the 28 (39%) patients who remained disease-free after surgery were high, 14 (50%) intermediate and 3 (11%) low risk according to the MSC test (Figure 3). The MSC test was already positive in 14 participants with a plasma sample available before diagnosis (median time = 1.1 years, IQR = 1.0). Considering the 25 high and intermediate risk subjects at diagnosis, reduction of MSC risk profile from high to low (*n* = 5), high to intermediate (*n* = 3) and intermediate to low (*n* = 11) was observed in 19 (76%) first post-surgery plasma sample (median time = 1.6 years, IQR = 1.4, *p* = 0.01). Of these 25 subjects none had an increase in the MSC risk profile from diagnosis to first post-surgery plasma sample. Of the 17 patients with a second post-operative plasma sample available (median time = 4.0 years, IQR = 3.3), a further reduction of risk profile was observed, being 76% low and 24% intermediate risk, while none remained high risk.

**Figure 4:**
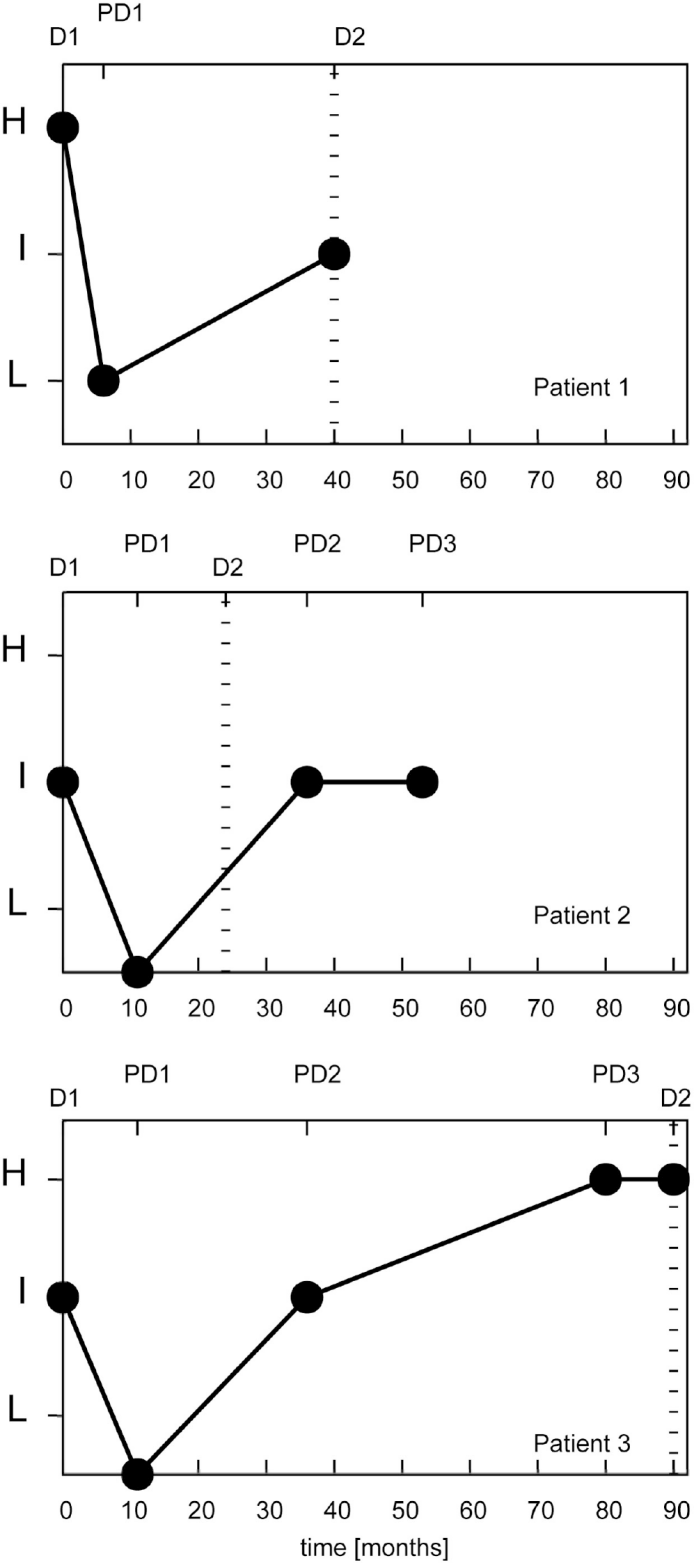
miRNA signature classifier risk profile (MSC) of 3 lung cancer patients with recurrent disease D1: miRNA signature at diagnosis; D2: time at second diagnosis with miRNA signature (if available); PD1, PD2, PD3: miRNA signature at first, second and third post-operative plasma sample, respectively. H: high; I: intermediate; L: low.

Of 3 patients with recurrent disease, a stage I adenocarcinoma (ADC) with high risk returned to low risk after curative surgery, but raised to intermediate risk when developed a second primary lung cancer 3 years later (Figure 4A). A second patient with a stage I ADC and an intermediate risk MSC decreased to low risk after surgery, while the MSC test on a plasma sample collected 3 years later, after radiotherapy to treat a lung metastasis to the brain, revealed an intermediate risk (Figure 4B). A third patient with a stage I ADC decreased from intermediate to low risk 10 months after surgery, then raised to intermediate 25 months later and finally to high risk 10 months before a lung nodule resection which at histopathological analysis resulted a metastatic site of a primitive breast cancer occurred 4 years before (Figure 4C).

## DISCUSSION

The present study provides further validation of the MSC test [18], a plasma-based miRNA test, and suggests its clinical utility as both prognostic and monitoring tool, alone as well as in combination with other clinico-pathological parameters. Analysis on 84 lung cancer patients identified in large LDCT screening cohorts (33402 person-years) revealed a very poor outcome of stage II–IV lung cancer cases and of interval tumors occurred among two rounds of screening.

The MSC analysis stratified the patients according to their survival, even after adjusting for tumor stage, and was able to refine prognosis within specific clinico-pathological groups. In particular, 20 out of 24 (83%) LDCT-detected patients who died were high risk according to the MSC test. Moreover, 63% of stage II–IV patients, as well as all 3 stage I patients who died at five years, were high risk highlighting the additional prognostic value of the MSC. On the other hand, patients with low or intermediate MSC risk were mostly (71%) stage I with a better and longer survival. If confirmed in larger series, the MSC test might be utilized for selecting patients with high risk profile who could benefit from adjuvant therapies irrespective of low stage disease.

Noteworthy, only 9 (11%) of the 84 patients were low risk MSC supporting the sensitivity of the MSC testing and the only patient who died was an interval cancer at stage IV that was also missed by LDCT imaging. On the other hand, MSC identified 7 of the 8 patients with interval cancer and a plasma sample available and 31 of the 35 (89%) stage II–IV patients. Combination of MSC and LDCT seems therefore effective to improve sensitivity of lung cancer screening.

Liquid biopsies-based biomarkers could be used to assess non-invasively the level of the individual risk. Our previous finding that MSC test identified intermediate or high risk subjects before lung cancer detection by LDCT [13, 18] supports our conclusion that the 24 miRNAs composing the MSC could be likely released from the damaged/diseased lung microenvironment, in particular from epithelial and/or stromal components which are strongly affected by smoking carcinogen exposure, signaling tumor onset and reflecting the tumor biological aggressiveness [22]. Indeed, several miRNA composing MSC control biological pathways highly relevant in lung cancer onset. mir-17, 19b, 92a, 106a are key oncogenic components of the oncomir clusters mir-17~92 and mir-106a-363, whose deregulation have been widely reported in numerous solid tumors, including lung [23] and also in regulation of response to microenvironmental toll-like receptor triggering [24]. Mir-197 and mir-660 target NOXA and MDM2 p53- related genes, respectively, and their replacement has been shown to achieve therapeutic effect in p53-wild type cancer [25, 26]. Mir-221 and mir-486-5p block PTEN expression leading to activation of the PTEN/AKT survival pathway [27]. Interestingly, other miRNAs of the MSC signature behave as general sensors of metabolic and stress related pathways such a mir-451 [28], a sensor of glucose levels which regulates LKB1/AMPK signaling and allows adaptation to metabolic stress and mir-486-5p, a cardiac/skeletal muscle enriched miRNA whose reduced expression in plasma may reflect cancer-induced skeletal muscle dysfunction and cachexia [29].

We present here the first evidence that the MSC test can be successfully employed to monitor the disease status at follow-up in LDCT screening detected lung cancer patients. The significant reduction of the MSC risk result after curative surgery observed in subjects who remained disease-free, together with the observation that in the three relapsing patients the MSC test returned to intermediate or high risk at the time of second primary or metastatic progression, were indications of the biological specificity of MSC test to lung cancer.

Conversely, a subset of subjects (24%) retained an elevated MSC profile even after cancer removal which could reflect the persistence a host/microenvironment–related risk profile. In this regard, the MSC could improve screening performance with LDCT by reducing further follow-up exams in low risk subjects while focusing efforts and resources towards subjects with an elevated risk after MSC analysis, who are potentially more likely to relapse.

In conclusion, this study shows that the MSC results obtained from lung cancer patients detected in screening programs with extended follow-up may be sensitive and accurate to improve individual risk assessment and may be adequate for clinical decision-making.

## PATIENTS AND METHODS

### Study population

In 2000 a five-year prospective pilot trial offering yearly LDCT to 1035 current or former heavy smokers volunteers with a smoking history of at least 20 pack-years, 50 years of age or older, was launched in Milan [19]. In 2005, the Multicentric Italian Lung Detection (MILD) trial was initiated and over the following 5 years it enrolled 4099 heavy-smoker volunteers with the same characteristics of the previous trial. Volunteers were randomized to a control arm and an early detection arm, the subjects of the latter group further randomized to receive annual or biennial LDCT [8]. Details of these screening programs were described elsewhere [8, 19].

Overall, between 2000 and 2010, a total of 3411 subjects underwent annual (2225) or biennial (1186) LDCT and 111 subjects developed lung cancer during the first five years of screening.

Plasma samples of 84 subjects with lung cancer collected at the time of disease detection were available to perform the MSC test. For 31 patients who underwent curative resection of their primary lung cancer multiple (*n* = 100) plasma samples were also suitable for MSC analysis.

All participants were followed up until January 2015. Median follow-up of the alive patients was 5.9 years (IQR = 3.8). The actuarial five-year overall survival according to clinical and pathological characteristics was calculated for the subset of 84 patients suitable for plasma MSC analysis, according to three different risk groups (high, intermediate or low) [18].

### MicroRNA profiling

Total RNA was extracted from 200 μl of plasma using the mirVana PARIS Kit (Life-Technologies) and eluted in 50 μl of buffer. MicroRNA expression was determined in 3 μl of eluted RNA using the Multiplex Pools Protocol on custom-made microfluidics card (Life-Technologies) as previously described [18, 20].

Patients were classified as low, intermediate or high risk MSC based on the algorithm generated from the combination of four different signatures as previously described [18] and summarized in Supplementary Table S1. Briefly, low risk MSC included patients negative for all signatures, whereas positive patients were classified as intermediate risk MSC when positive for risk of disease (RD) and/or presence of disease (PD) only, and as high risk MSC when at least one of the two signatures of aggressive disease (RAD, PAD) was positive.

### Statistical analysis

The continuous variables were given as mean values ± standard deviation (SD) and median with IQR. The categorical variables were analyzed using contingency table analysis and the Chi-squared test with Yates correction or Fisher's exact test, as appropriate. Survival curves were estimated using the Kaplan-Meier method and were compared by the log-rank test [21].

Among patients with a MSC risk profile at diagnosis and at first post-operative plasma sample, we tested the hypothesis that the reduction of the intermediate and high MSC risk profile (defined as a reduction level of at least one step) was statistically different from 50%. All tests were two-sided and *p*-values < 0.05 were considered statistically significant. Statistical analyses were performed using SAS 9.2 statistical software (SAS Institute, Cary, NC) and STATA statistical software (version 11; StataCorp, College Station, TX, USA).

## SUPPLEMENTARY TABLE


