# Correction: Circulating microRNA signature as liquid-biopsy to monitor lung cancer in low-dose computed tomography screening

**DOI:** 10.18632/oncotarget.27256

**Published:** 2019-10-15

**Authors:** Stefano Sestini, Mattia Boeri, Alfonso Marchiano, Giuseppe Pelosi, Carlotta Galeone, Carla Verri, Paola Suatoni, Nicola Sverzellati, Carlo La Vecchia, Gabriella Sozzi, Ugo Pastorino

**Affiliations:** ^1^ Unit of Thoracic Surgery, Fondazione IRCCS Istituto Nazionale Tumori, Milan, Italy; ^2^ Unit of Tumor Genomics, Department of Experimental Oncology, Fondazione IRCCS Istituto Nazionale Tumori, Milan, Italy; ^3^ Unit of Radiology, Fondazione IRCCS Istituto Nazionale Tumori, Milan, Italy; ^4^ Department of Pathology and Laboratory Medicine, Fondazione IRCCS Istituto Nazionale Tumori, Milan, Italy; ^5^ Department of Clinical and Biomedical Sciences Luigi Sacco, University of Milan, Milan, Italy; ^6^ Department of Statistics and Quantitative Methods, Division of Biostatistics, Epidemiology and Public Health, Laboratory of Healthcare Research and Pharmacoepidemiology, University of Milano-Bicocca, Milan, Italy; ^7^ Department of Clinical Sciences, Section of Radiology, University of Parma, Milan, Italy; ^8^ Department of Clinical Sciences and Community Health, University of Milan, Milan, Italy

**This article has been corrected:** In the Introduction section, the second sentence of the second paragraph was worded incorrectly. The proper sentence is as follows:

A significant reduction in lung cancer mortality was reported among the subjects enrolled in the LDCT arm of the National Lung Screening Trial (NLST) when compared to the chest X-ray arm, along with a false discovery rate of 96.4% and an overdiagnosis global rate of 18.5%, reaching 78.9% for indolent cancers in the LDCT arm.

Original article: Oncotarget. 2015; 6:32868–32877. 32868-32877. https://doi.org/10.18632/oncotarget.5210

