# Supplementary material for: Circulating microRNA signature as liquid-biopsy to monitor lung cancer in low-dose computed tomography screening
Source: Oncotarget. 2015 Oct 6;6(32):32868–77. doi: 10.18632/oncotarget.5210 (PMC4741735; doi:10.18632/oncotarget.5210)
Supplement: Supplementary file 1 [file oncotarget-06-32868-s001.pdf]

SUPPLEMENTARY TABLE

Supplementary Table S1: Algorithm for MSC risk level stratification

MSC	RD	PD	RAD	PAD
High risk	+/–	+/–	+	+/–
	+/–	+/–	+/–	+
Intermediate risk	+	+/–	–	–
	+/–	+	–	–
Low risk	–	–	–	–

RD: risk of disease; PD: presence of disease; RAD: risk of aggressive disease; PAD: presence of aggressive disease.
